# Magnetoencephalography as a Tool in Psychiatric Research: Current Status and Perspective

**DOI:** 10.1016/j.bpsc.2017.01.005

**Published:** 2017-04

**Authors:** Peter J. Uhlhaas, Peter Liddle, David E.J. Linden, Anna C. Nobre, Krish D. Singh, Joachim Gross

**Affiliations:** a Institute for Neuroscience and Psychology, University of Glasgow, Glasgow; b Division of Psychiatry and Applied Psychology, University of Nottingham, Nottingham; c MRC Centre for Neuropsychiatric Genetics and Genomics, School of Medicine, Cardiff University, Cardiff; d Cardiff University Brain Research Imaging Centre, School of Psychology, Cardiff University, Cardiff; e Oxford Centre for Human Brain Activity, University of Oxford, Oxford, United Kingdom; f Department of Experimental Psychology, University of Oxford, Oxford, United Kingdom

**Keywords:** Biomarker, Brain imaging, Magnetoencephalography, Neural oscillations, Psychiatry, Translational Research

## Abstract

The application of neuroimaging to provide mechanistic insights into circuit dysfunctions in major psychiatric conditions and the development of biomarkers are core challenges in current psychiatric research. We propose that recent technological and analytic advances in magnetoencephalography (MEG), a technique that allows measurement of neuronal events directly and noninvasively with millisecond resolution, provides novel opportunities to address these fundamental questions. Because of its potential in delineating normal and abnormal brain dynamics, we propose that MEG provides a crucial tool to advance our understanding of pathophysiological mechanisms of major neuropsychiatric conditions, such as schizophrenia, autism spectrum disorders, and the dementias. We summarize the mechanisms underlying the generation of MEG signals and the tools available to reconstruct generators and underlying networks using advanced source-reconstruction techniques. We then surveyed recent studies that have used MEG to examine aberrant rhythmic activity in neuropsychiatric disorders. This was followed by links with preclinical research that has highlighted possible neurobiological mechanisms, such as disturbances in excitation/inhibition parameters, that could account for measured changes in neural oscillations. Finally, we discuss challenges as well as novel methodological developments that could pave the way for widespread application of MEG in translational research with the aim of developing biomarkers for early detection and diagnosis.

Neuroimaging has played a fundamental role in psychiatric research in recent decades, shaping a new conceptualization of major brain disorders as impairments in neural circuits through the interrogation of the anatomical and functional architecture of large-scale networks (1, 2). These important insights have been largely supported through studies conducted with magnetic resonance imaging (MRI), a brain imaging technique with excellent spatial resolution, which also allows for functional imaging of neurovascular signals. However, research into the underlying pathophysiology of psychiatric conditions has been impeded by the fact that these imaging techniques allow only indirect links to cellular and physiological mechanisms underlying observed signal changes and thus only limited relationships to preclinical research. Moreover, MRI approaches are unable to capture neural processes with high temporal resolution, which is essential for measuring fast rhythmic fluctuations of neuronal events that have been recently implicated in major psychiatric conditions (3).

## Neuronal Dynamics in Large-Scale Networks

It is conceivable that mechanistic insights into circuit dysfunctions require a stronger focus on noninvasive techniques that allow a direct assessment of neuronal dynamics at high temporal resolution. This is because recent data highlight that cognitive and executive processes during normal brain functioning emerge from the coordinated activity of distributed neuronal populations that are dynamically configured on the backbone of anatomical connections (4, 5, 6). Measurements from invasive as well as noninvasive electrophysiology support this hypothesis through demonstrating close relationships between modulations in the amplitude and synchrony of rhythmic activity and task-dependent and state-dependent parameters and cognitive and perceptual processes (7, 8, 9).

In addition to the functional significance of such rhythmic fluctuations, much work has been devoted to the analysis of synaptic mechanisms and circuits that support the generation of oscillatory activity and its synchronization (10). Establishing relationships between levels of organization, from cellular physiology to large-scale networks, will be a fundamental prerequisite for translational research. Only with these links does it become possible to quantify neural measures in the human brain that can be compared with observations in animal models and patient data to discover pathophysiological mechanisms and to target intervention to correct circuit dysfunctions (3).

## Magnetoencephalography as a Tool in Brain Research

Electroencephalography (EEG) and magnetoencephalography (MEG) provide noninvasive measurements of fluctuations in the excitability of neuronal populations with high temporal resolution that can provide potential direct links with preclinical research. Although EEG has been widely applied in the investigation of psychiatric disorders for many decades, MEG has been applied only more recently in the identification of pathophysiological mechanisms.

**Figure 1:**
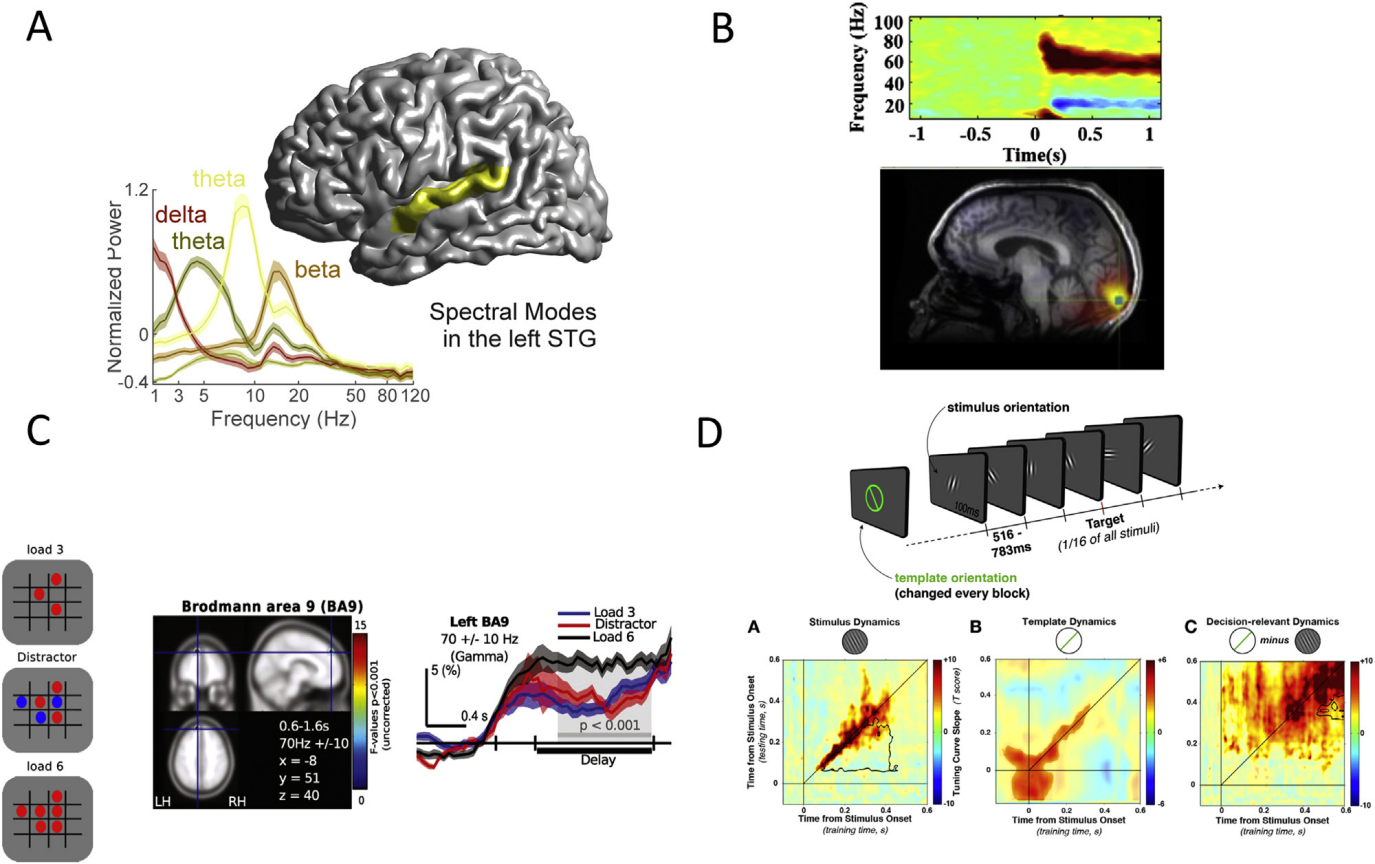
Overview of current magnetoencephalography findings during normal brain functioning. **(A)** Analysis of magnetoencephalography resting-state data in combination with source localization reveals that individual brain areas are characterized by a specific “spectral fingerprint” consisting of a mixture of brain rhythms (example shown here is from the left superior temporal gyrus [STG]). **(B)** Onset of a moving grating pattern induces strong rhythmic brain activity in visual areas in the gamma frequency range between 40 and 100 Hz. **(C)** Gamma band oscillations in prefrontal cortex predict working memory. (Left panel) Visuospatial working memory task. (Middle panel) The 60- to 80-Hz activity (0.6–1.6 seconds) across task conditions during the delay period for the left Brodmann area 9 (BA9) displayed on axial, sagittal, and coronal sectional views of the Montreal Neurological Institute template brain. (Right panel) Time course of 60- to 80-Hz activity for peak voxels averaged across trials in BA9. The light gray region corresponds to the temporal interval of significant differences between conditions (*p* < .001; corrected; post hoc *t* test). In BA9, there was a significant increase of 60- to 80-Hz activity from 0.6 to 1.6 seconds during load 6 compared with load 3 and distractor conditions, while activity during load 3 and the distractor condition was similar. **(D)** In this study, participants had to identify a template orientation in a stream of stimuli. Decoding of magnetoencephalography signals allowed the separation of stimulus-related (left) and template-related (right) information. [**(A)** Adapted with permission from Keitel and Gross (96); **(B)** modified with permission from Muthukumaraswamy and Singh (12); **(C)** adapted with permission from Roux *et al.* (8); **(D)** modified with permission from Myers *et al.* (97).]

Both MEG and EEG record a signal that is based primarily on postsynaptic potentials of pyramidal cells (11). However, there are important differences between both techniques (Table 1). MEG measures magnetic fields, whereas EEG measures electric potentials at the scalp. Electric signals are significantly distorted as they pass through different tissues with varying conductivities (cerebrospinal fluid, skull, skin). In contrast, magnetic fields are hardly affected by different tissues, which leads, for example, to improved estimates of high-frequency oscillations (Figure 1B) (12). Moreover, localization of generators of neural activity is more accurate for MEG than for EEG—especially if the complex geometries of these different tissues are not modeled correctly (13). Another difference between MEG and EEG is their sensitivity to the spatial orientation of the underlying generators (14). Whereas MEG is largely insensitive to radial sources that point toward the center of the head or away from it, EEG is sensitive to all orientations, although the amount of cortex truly silent to MEG may be relatively small (15). It should also be noted that EEG records signals relative to a reference electrode, the definition of which requires adequate consideration. In contrast, MEG recordings are reference free. MEG is also less susceptible to contamination by muscle artifacts, particularly those that result from volume conduction across electrodes, including the reference (16).

Optimal use of the rich information content of MEG signals often benefits from reconstruction of the generators of these signals. Source reconstruction relies on the combination of information represented in different MEG channels and combines with volume conductor models typically derived from individual anatomical MRI scans to compute tomographic estimates of activation on a three-dimensional grid covering the whole brain or the cortical surface. Source reconstruction has several benefits. It increases the signal-to-noise ratio, and it leads to more interpretable results because it reveals the location of the generators underlying the measured MEG signals (17). However, the sensitivity of MEG systems depends not only on the orientation of the generator but also on its location. MEG sensors are more sensitive to cortical generators than to subcortical generators (18).

## MEG Studies During Normal Brain Functioning

### Neural Oscillations During Cognitive Processes

Until recently, the noninvasive measurement of rhythmic activity in cognitive neuroscience has largely relied on sensor or electrode estimates of neural activity that provided correlative evidence with cognitive processes. Advances in MEG approaches have now allowed for the noninvasive mapping of the neuronal dynamics in large-scale networks, which have provided novel insights into the role of neural oscillations during perceptual and higher cognitive processes (19). Importantly for clinical research and development of biomarkers, such networks can be recreated in MEG measurements with high test-retest reliability (17, 20).

Recent MEG studies have shown that top-down, attention-related signals are critical to the selection and integration of information that is relevant given the goals of an individual in a particular context (21). These biasing signals are readily measured using MEG, and it is possible to identify the sources of attention-related control in the distributed network of high-level associative areas and their modulatory consequences in perceptual areas (22, 23). MEG studies have also contributed to our understanding of how attentional control and modulation guide perception of individuals (24) as well as how individuals flexibly focus on specific contents within memory (8, 25). These processes of dynamic prioritization and selective routing of information processing are ubiquitous to healthy brain function and cognition (26), and their disruption is a major factor in many neuropsychiatric conditions (27). Importantly, MEG data have sufficient information content to allow decoding of cognitive states (Figure 1C) (8), highlighting the mechanistic involvement of MEG-measured oscillations in cognitive processes. Finally, emerging data suggest close relationships between rhythmic activity as assessed with MEG and the anatomical expression profile of neural oscillations (28) as well as with spectral characteristics of local field potentials from invasive electrophysiology (29), raising the exciting prospect of measuring physiologically realistic neuronal dynamics in extended networks.

### Resting-State Networks

Recent applications of MEG have led to novel insights into the complex dynamics of resting-state networks, which are an important target for psychiatric research (30). The networks disclosed by MEG-informed source reconstruction closely resemble the resting-state networks delineated using functional MRI (fMRI) (31), such as the default mode network, dorsal attention networks, and motor networks, but provide additional information on the contribution of distinct frequencies to the organization of the dynamic connectome (32). The high temporal resolution of MEG in principle allows many different approaches to quantifying coupling between spatially distinct brain regions, including power-power coupling, phase-phase correlations, and cross-frequency phase-amplitude coupling. Recent work has also extended the application of MEG to the investigation of cortical-subcortical networks during rest. Contributions from deeper sources should be detectable in MEG data, given that their fields are strong enough to propagate to the sensor array of MEG superconducting quantum interference devices (33). In a recent study, Roux *et al.* (34) employed MEG to investigate interactions between neocortical gamma band activity and thalamic alpha oscillations, which are consistent with recent theoretical and empirical data that highlight the role of phasic inhibition in the coordination of cortical activity. Similar data on the possibility to detect deep structures have been obtained for hippocampal theta band activity (35).

## MEG Studies in Neuropsychiatric Disorders

In addition to novel insights into the role and organization of rhythmic activity and its relationship to normal cognitive processes, MEG has also been increasingly applied toward identifying alterations in neural oscillations in several psychiatric conditions, such as schizophrenia (ScZ), autism spectrum disorder (ASD), and dementia. These studies have provided novel evidence on the importance of alterations in neuronal dynamics in psychiatric conditions.

### Schizophrenia

A substantial body of EEG/MEG studies support the hypothesis that ScZ is associated with impaired neural oscillations and their synchronization (36), which is consistent with evidence highlighting disturbances in gamma-aminobutyric acidergic interneurons and/or *N*-methyl-D-aspartate receptors in the disorder (37, 38). Until recently, the large body of work implicating abnormal gamma band activity in ScZ has focused on rhythmic activity in the 40-Hz frequency range with EEG. The most consistent evidence from these studies is a reduction in both amplitude and phase locking of 40-Hz auditory steady-state responses (39, 40).

**Figure 2:**
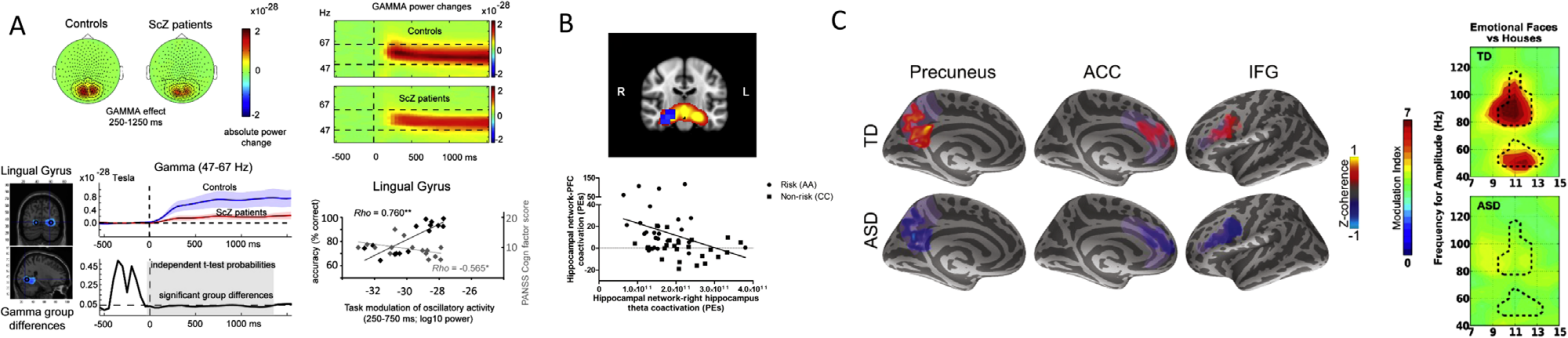
Overview of magnetoencephalography (MEG) findings in neuropsychiatric disorders. **(A)** High-frequency oscillations in schizophrenia (ScZ). MEG data from 16 patients with chronic ScZ during a visual attention task in which participants are required to detect a speed change of a sine-wave grating (46). (Top panels) Time frequency representations for sensor-level MEG data indicating a significant reduction in 45- to 70-Hz power in patients with ScZ relative to control subjects. (Bottom panels) Source space analysis and virtual channel time course in the lingual gyrus (left panel). Downregulation of 45- to 70-Hz spectral in patients with ScZ correlates significantly with decreased detection rates as well as elevated ratings on the Positive and Negative Syndrome Scale Cognitive (PANSS Cogn.) factor. **(B)** Hippocampal theta is modulated by *ZNF804A* genotype. (Top panel) Risk allele homozygotes show decreased coactivation of the right hippocampus in the theta band (shown in blue) (*p* < .05 after familywise error correction for multiple comparisons; *n* = 525 per group) compared with the rest of the hippocampal network (red heat map thresholded at 3 > *Z* > 8) vs. nonrisk homozygotes. (Bottom panel) Intrahippocampal theta and hippocampal–prefrontal cortex (PFC) coactivation are inversely related (Spearman rho = .40; *p* = .005; *n* = 25). Correlations are negative within both groups (risk [circles]: Spearman rho = .23; nonrisk [squares]: Spearman rho = .21). **(C)** Long-range functional connectivity in autism spectrum disorder (ASD). Source space coherence for emotional faces normalized by coherence for houses between the fusiform face area and the rest of the cortex for the typically developing (TD) group (upper panel) and ASD group (lower panel). The *Z* coherence values shown are masked by the three clusters that showed statistically significant differences (*p* < .05 corrected) between the groups (i.e., all values not within these clusters were set to 0). The significant clusters overlap with precuneus, anterior cingulate cortex (ACC), and inferior frontal gyrus (IFG) anatomical labels from FreeSurfer (purple shading). For each cluster, the maps are shown at the time of maximal group difference. (Right panel) Phase-amplitude coherence values for emotional faces normalized by phase-amplitude coherence for houses for the TD group (upper panel) and ASD group (lower panel). Dotted lines indicate significant group differences in phase-amplitude coherence for houses. L, left; R, right. [**(A)** Adapted with permission from Grent-’t-Jong *et al.* (47); **(B)** adapted with permission from Cousijn *et al*. (51); **(C)** adapted with permission from Khan *et al.* (58).] AA, “risk” homozygotes; CC, "nonrisk” homozygotes; PE, parameter estimates.

MEG studies that examined auditory steady-state responses at both 80 Hz and lower frequency stimulation (41, 42) suggested that dysfunctions in entrainment may not be specific to the 40-Hz frequency range. Consistent with these findings, Grützner *et al.* (43) and Sun *et al.* (44) have revisited the role of high gamma band oscillations in ScZ and their relationship to perceptual impairments. MEG data from invasive recordings in monkeys and intracranial EEG recordings in humans suggest that gamma band oscillations encompass a much wider frequency range of up to 200 Hz. This so-called high gamma band activity (60–200 Hz) may be important for cortical computations (45) and can be measured with a high signal-to-noise ratio in MEG recordings (46). MEG data from both patients with chronic ScZ (Figure 2A) (44, 47) and unmedicated patients experiencing a first episode of ScZ (43) showed a widespread deficit in the power of gamma band oscillations between 60 Hz and 120 Hz that were associated with large effect sizes.

Extensive evidence from fMRI and other modalities supports the hypothesis that disordered connectivity between spatially distinct brain regions plays a core role in ScZ (48). With its capacity to measure the temporal dynamics of connectivity on a much shorter time scale than fMRI, MEG has the potential to expand the understanding of disturbances in connectivity (49). So far there have been few attempts to fully exploit the temporal resolution of MEG in the measurement of functional connectivity. Liddle *et al.* (50) demonstrated aberrant beta band signaling during a salience task, which suggests reduced coupling between spectral power in the insula and gamma band activity in the visual cortex of patients with ScZ. In addition, Cousijn *et al.* (51) examined hippocampus–prefrontal cortex functional connectivity in healthy volunteers in relationship to the common variant on the *ZNF804A* gene that shows a genome-wide significant association with psychotic disorders (Figure 2B). A significant decrease in intrahippocampal theta activity as well as decreased hippocampus–prefrontal cortex connectivity identified with fMRI was observed, which is in agreement with findings on the effects of common risk variants on hippocampus–prefrontal cortex networks.

### Autism Spectrum Disorder

Similar to recent work in ScZ, establishing the contribution of aberrant neuronal dynamics to the pathophysiology of ASD has received considerable interest as preclinical evidence points toward a fundamental disturbance in excitation-inhibition balance parameters (52). This is consistent with the view that both ScZ and ASD are neurodevelopmental disorders, albeit with different developmental trajectories, that fundamentally involve disturbance in the maturation of cellular parameters ensuring effective neuronal inhibition (53).

This hypothesis is supported by emerging evidence from MEG that entrainment to 40-Hz stimulation in auditory cortices in ASD is impaired (54), replicating evidence from observations in patients with ScZ (see Schizophrenia above) (39). In addition, ASD is characterized by pronounced impairments in high-frequency oscillations in the 60- to 120-Hz frequency range during complex visual processing (55), which is accompanied by reduced long-range synchronization (56), indicating a fundamental disruption of large-scale networks that potentially can explain perceptual biases of local over global information (57). Further evidence for this hypothesis was reported by Khan *et al.* (58), who examined local and long-range cortical functional connectivity between and within the fusiform face area and the precuneus, inferior frontal gyrus, and anterior cingulate cortex (Figure 2C). Participants with ASD were characterized by reduced alpha-gamma coupling within the fusiform face area and reduced long-range coherence with the precuneus, inferior frontal gyrus, and anterior cingulate cortex. Taken together, these results suggest that failure to entrain neuronal assemblies both within and across cortical regions may be characteristic of ASD, consistent with recent pathophysiological theories of ASD postulating aberrant large-scale organization of functional networks (59).

### Alzheimer’s Disease

Identification of noninvasive biomarkers for the diagnosis and early detection of neurodegenerative disorders, such as Alzheimer’s disease (AD), is a major challenge for current psychiatric and neurological research. In the field of AD research, MEG has been mainly applied to investigate alterations in functional and resting-state networks (60).

Consistent with EEG data, MEG-measured resting-state neural oscillations in AD are characterized by a reduction of rhythmic activity at alpha/beta band frequencies, while the contribution of slower rhythms to the power spectra is increased (61). More recently, several groups have examined the organization of resting-state networks in combination with advanced analytic approaches, such as graph theory (62). Patients with AD are characterized by decreased network connectivity in the alpha band range, suggesting that highly connected neural network “hubs” may be especially at risk in AD. Reductions in functional connectivity in the alpha band range may be related to cognitive deficits and disease severity as indicated by correlations between loss of interregional connectivity and impairments in executive control, episodic memory, and visuospatial processing (63). However, these findings are not specific to AD, as similar impairments have been observed in ScZ (64).

The early identification of individuals with mild cognitive impairment (MCI), an intermediate stage between normal cognitive aging and dementia, is of major clinical importance. In this context, reliable biomarkers that allow prediction of conversion to AD are crucial because longitudinal studies have found that the conversion rate from MCI to AD is only 10% to 15% per year (65). López *et al.* (66) compared individuals with MCI who progressed to overt AD over a mean duration of 1 year with individuals with MCI who did not progress to dementia. The individuals with progressive MCI exhibited hyperconnectivity in the alpha band between anterior and posterior brain regions. This appears paradoxical in light of the evidence discussed above indicating decreased alpha band connectivity in established AD. However, the finding of López *et al.* is consistent with EEG evidence (67, 68) that has highlighted increased coherence values at alpha as well as delta and theta frequencies in individuals with MCI who have converted to AD compared with nonconverters.

There is also preliminary evidence from task-related MEG data that alterations of spontaneous neural oscillations extend to recruitment of functional networks involved in memory processes. Individuals with MCI are characterized by reductions in high-frequency activity as well as synchronization during a Sternberg task, while lower frequencies, such as at theta frequencies, are increased. However, further studies are required to determine the utility of MEG-measured neural oscillations as biomarkers for prediction of AD and characterization of the underlying networks (69).

## MEG and Translational Neuroimaging

One of the attractions of MEG for translational research is that the signals it measures can be compared with analogous measures derived from invasive electrophysiology in animals or humans. First attempts have been made at identifying correlates of MEG signals in invasive recordings (70). Such an integration and cross-validation of noninvasive and invasive data, which are several orders of magnitude superior in spatial resolution and are more amenable to a mechanistic interpretation, is particularly useful for translation in clinical disorders. This program is probably most advanced in the field of movement disorders, particularly Parkinson’s disease, because both animal and human invasive recordings in the context of deep brain stimulation are available. A large body of invasive neurophysiology literature in the monkey model has implicated aberrant beta oscillations (71, 72), and similar observations have been made in humans with both invasive and MEG recordings (73).

For AD, several rodent models are based on genetic variants that produce forms of early-onset dementia with Mendelian inheritance (74, 75). Preliminary evidence from double transgenic mouse (with mutations on the genes for the amyloid precursor protein and presenilin 1) suggests hyperactivity of cortical neurons as a potential pathophysiological mechanism of AD (76), which could lead to impaired interneuron functions and aberrant gamma band oscillations (77). Although local hyperactivity has also been observed in some human studies, particularly in functional imaging studies with participants at risk of developing AD (78, 79, 80), more work is needed to enable the comparability between these very different scales of analysis. MEG in participants at high genetic risk for AD can play a crucial role in this respect because it allows for comparison with electrophysiological parameters derived from rodent models with homologous genetic variants.

Similar opportunities for translation between human and animal electrophysiology are emerging for neurodevelopmental disorders. Recent bioinformatics analyses of pathways associated with both rare (e.g., pathogenic copy number variants) and common risk variants for ScZ have highlighted the postsynaptic density, whose integrity is crucial for *N*-methyl-D-aspartate receptor function, and the gamma-aminobutyric acidergic inhibitory system (81, 82). These neurotransmitter systems play a central role in the control of neural oscillations (10), and the combination of invasive (in animal models) and noninvasive (MEG, EEG) neurophysiology thus opens up attractive opportunities for translational research (3). MEG findings from participants with risk copy number variants for ScZ and/or autism have only recently started to emerge. One study reported that the microdeletion at 16p11.2 (which confers risk for autism), but not the corresponding microduplication (which confers risk for autism and ScZ), was associated with a delayed auditory M100 response (83). Although no direct comparison with animal electrophysiology is available, initial work in the mouse model of 16p11.2 has included in vitro electrophysiology, demonstrating changes in the excitatory postsynaptic potentials in medium spiny neurons of the nucleus accumbens (84). It would be desirable to move on to projects using analogous paradigms in human and animal carriers of the same copy number variant to enhance the bidirectional translation of these findings.

## Toward MEG Biomarkers

Given the physiological plausibility of MEG signals and their high dimensionality, MEG data may be considered ideal for the development of biomarkers in psychiatry. One obstacle to this goal is the overlap of changes in neural oscillations across different disorders as highlighted in this review. While similarities in neurodynamic signatures between different psychiatric disorders can be expected based on shared pathophysiological pathways (3), the current nonspecificity of the majority of MEG parameters as well as other neuroimaging measures is a serious challenge. Accordingly, it is important for current and future research to fully exploit the rich spectrum of MEG data that go beyond circumscribed descriptions of amplitude fluctuations in given frequency bands and brain regions. This would also facilitate the application of advanced machine learning algorithms that are ideally suited for high-dimensional data and that could eventually yield novel biotypes based on alterations of the temporospatial patterning of large-scale dynamics [for a recent application of such approach in ScZ, see (85,86)]. Such “biotypes” could then be employed for subtyping of currently heterogeneous syndromes and ultimately allow novel insights into neurobiological mechanisms.

The search for mechanistic interpretations of alterations in neuronal dynamics in psychiatric syndromes could also be facilitated by integration of MEG data with advances in computational modeling of whole-brain networks (87). These computational models have recently begun to incorporate increasingly realistic simulations of physiological parameters that would allow the testing of pathophysiological hypotheses in silico (88).

## Methodological and Analytic Innovations in MEG Research

The limited application of MEG in psychiatric research so far has been due to the small number of MEG systems compared with MRI and EEG, the complexity of data analysis, limited resolution for deeper brain structures, and high maintenance costs. Recent developments have addressed several of these issues, which we believe will significantly enhance the impact of MEG for translational research in psychiatry.

Until recently, MEG systems required regular helium supplies leading to considerable operating costs. However, advances in helium recycling technology have led to dramatic reductions in helium usage. Moreover, novel MEG sensors that can operate at room temperatures have been developed and offer the promise of conducting MEG measurements without the confines of elaborate dewar and cooling systems (89). Together, these developments will significantly enhance the availability of MEG systems through reducing running costs, which could lead to more widespread application of MEG in basic and clinical research.

For the analysis of MEG signals, several open-source software packages have been developed in recent years that provide standardized and state-of-the-art analytic tools for analysis of MEG data. Applications, such as Fieldtrip (90), Brainstorm (91), and statistical parametric mapping, offer a wealth of methods for time-frequency analysis, source reconstruction, and connectivity measures that have significantly improved the standards of data processing and analysis in the MEG community. This development has been accompanied by guidelines for conducting and reporting MEG research (92).

In addition, a number of recent methodological developments have seen a significant improvement in spatial localization of MEG signals. Until recently, estimates of neural generators in MEG data have been in the centimeter range and largely confined to cortical sources. As emphasized in this review, our data (34) and those of others (33) suggest that deep sources, such as the thalamus and hippocampus, can be reliably detected given that certain conditions, such as trial numbers, are met, significantly extending the application of MEG to the investigation of subcortical networks. Moreover, in recent work by Troebinger *et al.* (93), subject-specific head casts produced using three-dimensional printing technology improved localization and signal quality significantly over conventional strategies, and such head casts may ultimately allow for the differentiation between superficial and deep cortical laminae (94). Finally, emerging evidence suggests that brain stimulation techniques, such as transcranial direct current stimulation, can be applied during MEG measurements despite the considerable technical challenges (95), highlighting the possibility of establishing causal relationships between frequency-specific entrainment of neural oscillations and behavior and cognition.

Furthermore, recent studies have explored the suitability of conducting multisite MEG recordings and the integration of complex data sets into standardized analysis pipelines. The possibility of performing acquisition and analysis of MEG data across centers is an important issue for studies of the development of biomarkers for complex neuropsychiatric conditions, as the recruitment of large numbers of participants is frequently challenging for single academic sites. To address this challenge, the United Kingdom Medical Research Council has recently funded a unique collaborative project “Building Multi-site Clinical Research Capacity in Magnetoencephalography (MEG),” which aims to establish a database of MEG recordings from eight academic centers in the United Kingdom. Our preliminary data from several different tasks show that MEG data from different recording systems can be integrated into a single analysis platform, which shows very similar and compatible results. Accordingly, these findings provide strong groundwork for applying MEG in future multisite, clinical studies.

## Conclusions

The current review suggests that MEG has significant potential to provide a powerful tool for translational psychiatric research to discover pathophysiological mechanisms and biomarkers for early detection and diagnosis that will complement the current research conducted with fMRI or MRI. MEG allows the precise measurement of direct physiological processes combined with accurate reconstruction of underlying generators, which enable for the first time the measurement of neuronal dynamics on realistic time scales in large-scale networks. Given the emerging evidence that the origin of major syndromes, such as ScZ, ASD, and dementia, involves a fundamental disruption in the organization and coordination of such processes, we believe that MEG will provide a crucial tool for the advancement of translational research in psychiatry.

## Figures and Tables

**Table 1:** Comparison of EEG and MEG

Parameter	EEG	MEG	Comment
High-Frequency Oscillations	Fair SNR	Excellent SNR	MEG has improved SNR for gamma band activity compared with EEG (11).
Deep Sources	Good detection	Good evidence	EEG signals have a stronger contribution of deeper sources. However, emerging evidence suggests that MEG is also sensitive to activity in deeper structures (34, 35).
Source Orientation	Tangential/radial	Tangential	MEG is largely insensitive to radial sources, whereas EEG is sensitive to all orientations, although the amount of cortex truly silent to MEG may be relatively small (14).
Spatial Resolution	Centimeter range	Centimeter range	EEG and MEG have a spatial resolution of sources in centimeter range with MEG allowing for improved localization accuracy (13) and even mm accuracy (93). There is preliminary evidence for MEG to detect layer-specific activity (28). For EEG, source localization is also complicated by more complex volume conduction models.
Artifacts	Muscular, cardiac, ocular	Muscular, cardiac, ocular	EEG and MEG signals are contaminated by similar muscular, cardiac, and ocular artifacts. Separation between neuronal vs. non-neuronal signal in MEG data is facilitated by a reference-free recording and less contribution of myographic signals.
Availability/Costs	Widely available/low costs	Few recording devices/high costs	EEG systems are currently more widely available than MEG systems, and both initial acquisition and maintenance costs for MEG are significantly higher compared with EEG. However, in the next few years, advances in new MEG sensors will likely reduce costs for MEG systems and possibly increase availability.
Tolerability/Practicality	Well tolerated	Well tolerated	Preparation time for EEG recording is longer compared with MEG, and impedances may change over the course of recordings. MEG is more strongly affected by movement artifacts. Both techniques are well tolerated by patients.
Multimodal Brain Imaging	EEG allows for parallel application of a variety of brain stimulation approaches and fMRI	Combination with additional, concurrent brain imaging techniques is more challenging	There is emerging evidence that brain stimulation approaches, such tDCS and tACS, can now also be applied during MEG recordings (95).

EEG, electroencephalography; fMRI, functional magnetic resonance imaging; MEG, magnetoencephalography; mm, millimeter; SNR, signal-to-noise ratio; tACS, transcranial alternating current stimulation; tDCS, transcranial direct current stimulation.

## References

[1] Linden D.E. (2012). The challenges and promise of neuroimaging in psychiatry. Neuron.

[2] Buckholtz J.W., Meyer-Lindenberg A. (2012). Psychopathology and the human connectome: Toward a transdiagnostic model of risk for mental illness. Neuron.

[3] Uhlhaas P.J., Singer W. (2012). Neuronal dynamics and neuropsychiatric disorders: Toward a translational paradigm for dysfunctional large-scale networks. Neuron.

[4] Singer W. (2013). Cortical dynamics revisited. Trends Cogn Sci.

[5] Buzsaki G. (2006). Rhythms of the Brain.

[6] Von der Malsburg C., Phillips W.A., Singer W. (2010). Dynamic Coordination in the Brain. Strüngmann Forum Report.

[7] Gray C.M., König P., Engel A.K., Singer W. (1989). Oscillatory responses in cat visual cortex exhibit inter-columnar synchronization which reflects global stimulus properties. Nature.

[8] Roux F., Wibral M., Mohr H.M., Singer W., Uhlhaas P.J. (2012). Gamma-band activity in human prefrontal cortex codes for the number of relevant items maintained in working memory. J Neurosci.

[9] Helfrich R.F., Knepper H., Nolte G., Strüber D., Rach S., Herrmann C.S. (2014). Selective modulation of interhemispheric functional connectivity by HD-tACS shapes perception. PLoS Biol.

[10] Wang X.J. (2010). Neurophysiological and computational principles of cortical rhythms in cognition. Physiol Rev.

[11] Lopes da Silva F (2013). EEG and MEG: Relevance to neuroscience. Neuron.

[12] Muthukumaraswamy S.D., Singh K.D. (2013). Visual gamma oscillations: The effects of stimulus type, visual field coverage and stimulus motion on MEG and EEG recordings. Neuroimage.

[13] Vorwerk J., Cho J.H., Rampp S., Hamer H., Knösche T.R., Wolters C.H. (2014). A guideline for head volume conductor modeling in EEG and MEG. Neuroimage.

[14] Sarvas J. (1987). Basic mathematical and electromagnetic concepts of the biomagnetic inverse problem. Phys Med Biol.

[15] Hillebrand A., Barnes G.R. (2002). A quantitative assessment of the sensitivity of whole-head MEG to activity in the adult human cortex. Neuroimage.

[16] Yuval-Greenberg S., Tomer O., Keren A.S., Nelken I., Deouell L.Y. (2008). Transient induced gamma-band response in EEG as a manifestation of miniature saccades. Neuron.

[17] Tan H.R., Gross J., Uhlhaas P.J. (2015). MEG-measured auditory steady-state oscillations show high test-retest reliability: A sensor and source-space analysis. Neuroimage.

[18] Boesveldt S., Stam C.J., Knol D.L., Verbunt J.P., Berendse H.W. (2009). Advanced time-series analysis of MEG data as a method to explore olfactory function in healthy controls and Parkinson’s disease patients. Hum Brain Mapp.

[19] Palva S., Palva J.M. (2012). Discovering oscillatory interaction networks with M/EEG: challenges and breakthroughs. Trends Cogn Sci.

[20] Muthukumaraswamy S.D., Singh K.D., Swettenham J.B., Jones D.K. (2010). Visual gamma oscillations and evoked responses: Variability, repeatability and structural MRI correlates. Neuroimage.

[21] Nobre A.C.K., Kastner S., Nobre A.C.K., Kastner S. (2014). Attention: Time capsule 2013. The Oxford Handbook of Attention.

[22] Siegel M., Donner T.H., Oostenveld R., Fries P., Engel A.K. (2008). Neuronal synchronization along the dorsal visual pathway reflects the focus of spatial attention. Neuron.

[23] Gross J., Schmitz F., Schnitzler I., Kessler K., Shapiro K., Hommel B., Schnitzler A. (2004). Modulation of long-range neural synchrony reflects temporal limitations of visual attention in humans. Proc Natl Acad Sci U S A.

[24] Todorovic A., Schoffelen J.M., van Ede F., Maris E., de Lange F.P. (2015). Temporal expectation and attention jointly modulate auditory oscillatory activity in the beta band. PLoS One.

[25] Wallis G., Stokes M., Cousijn H., Woolrich M., Nobre A.C. (2015). Frontoparietal and cingulo-opercular networks play dissociable roles in control of working memory. J Cogn Neurosci.

[26] Engel A.K., Fries P., Singer W. (2001). Dynamic predictions: Oscillations and synchrony in top-down processing. Nat Rev Neurosci.

[27] Arnsten A.F., Rubia K. (2012). Neurobiological circuits regulating attention, cognitive control, motivation, and emotion: Disruptions in neurodevelopmental psychiatric disorders. J Am Acad Child Adolesc Psychiatry.

[28] Michalareas G., Vezoli J., van Pelt S., Schoffelen J.M., Kennedy H., Fries P. (2016). Alpha-beta and gamma rhythms subserve feedback and feedforward influences among human visual cortical areas. Neuron.

[29] Dalal S.S., Baillet S., Adam C., Ducorps A., Schwartz D., Jerbi K. (2009). Simultaneous MEG and intracranial EEG recordings during attentive reading. Neuroimage.

[30] Woodward N.D., Cascio C.J. (2015). Resting-state functional connectivity in psychiatric disorders. JAMA Psychiatry.

[31] Brookes M.J., Woolrich M., Luckhoo H., Price D., Hale J.R., Stephenson M.C. (2011). Investigating the electrophysiological basis of resting state networks using magnetoencephalography. Proc Natl Acad Sci U S A.

[32] Hipp J.F., Siegel M. (2015). BOLD fMRI correlation reflects frequency-specific neuronal correlation. Curr Biol.

[33] Attal Y., Bhattacharjee M., Yelnik J., Cottereau B., Lefèvre J., Okada Y. (2007). Modeling and detecting deep brain activity with MEG & EEG. Conf Proc IEEE Eng Med Biol Soc.

[34] Roux F., Wibral M., Singer W., Aru J., Uhlhaas P.J. (2013). The phase of thalamic alpha activity modulates cortical gamma-band activity: Evidence from resting-state MEG recordings. J Neurosci.

[35] Kaplan R., Horner A.J., Bandettini P.A., Doeller C.F., Burgess N. (2014). Medial prefrontal theta phase coupling during spatial memory retrieval. Hippocampus.

[36] Uhlhaas P.J., Singer W. (2010). Abnormal neural oscillations and synchrony in schizophrenia. Nat Rev Neurosci.

[37] Lewis D.A., Curley A.A., Glausier J.R., Volk D.W. (2012). Cortical parvalbumin interneurons and cognitive dysfunction in schizophrenia. Trends Neurosci.

[38] Kantrowitz J.T., Javitt D.C. (2010). N-methyl-d-aspartate (NMDA) receptor dysfunction or dysregulation: The final common pathway on the road to schizophrenia?. Brain Res Bull.

[39] Brenner C.A., Krishnan G.P., Vohs J.L., Ahn W.Y., Hetrick W.P., Morzorati S.L., O’Donnell B.F. (2009). Steady state responses: Electrophysiological assessment of sensory function in schizophrenia. Schizophr Bull.

[40] Thune H., Recasens M., Uhlhaas P.J. (2016). The 40-Hz auditory steady-state response in patients with schizophrenia: A meta-analysis. JAMA Psychiatry.

[41] Tsuchimoto R., Kanba S., Hirano S., Oribe N., Ueno T., Hirano Y. (2011). Reduced high and low frequency gamma synchronization in patients with chronic schizophrenia. Schizophr Res.

[42] Hamm J.P., Gilmore C.S., Picchetti N.A., Sponheim S.R., Clementz B.A. (2011). Abnormalities of neuronal oscillations and temporal integration to low- and high-frequency auditory stimulation in schizophrenia. Biol Psychiatry.

[43] Grützner C., Wibral M., Sun L., Rivolta D., Singer W., Maurer K., Uhlhaas P.J. (2013). Deficits in high- (>60 Hz) gamma-band oscillations during visual processing in schizophrenia. Front Hum Neurosci.

[44] Sun L., Castellanos N., Grützner C., Koethe D., Rivolta D., Wibral M. (2013). Evidence for dysregulated high-frequency oscillations during sensory processing in medication-naive, first episode schizophrenia. Schizophr Res.

[45] Morgan H.M., Muthukumaraswamy S.D., Hibbs C.S., Shapiro K.L., Bracewell R.M., Singh K.D., Linden D.E. (2011). Feature integration in visual working memory: parietal gamma activity is related to cognitive coordination. J Neurophysiol.

[46] Hoogenboom N., Schoffelen J.M., Oostenveld R., Parkes L.M., Fries P. (2006). Localizing human visual gamma-band activity in frequency, time and space. Neuroimage.

[47] Grent-’t-Jong T., Rivolta D., Sauer A., Grube M., Singer W., Wibral M., Uhlhaas P.J. (2016). MEG-measured visually induced gamma-band oscillations in chronic schizophrenia: Evidence for impaired generation of rhythmic activity in ventral stream regions. Schizophr Res.

[48] Friston K.J., Frith C.D. (1995). Schizophrenia: A disconnection syndrome?. Clin Neurosci.

[49] Schoffelen J.M., Gross J. (2009). Source connectivity analysis with MEG and EEG. Hum Brain Mapp.

[50] Liddle E.B., Price D., Palaniyappan L., Brookes M.J., Robson S.E., Hall E.L. (2016). Abnormal salience signaling in schizophrenia: The role of integrative beta oscillations. Hum Brain Mapp.

[51] Cousijn H., Tunbridge E.M., Rolinski M., Wallis G., Colclough G.L., Woolrich M.W. (2015). Modulation of hippocampal theta and hippocampal-prefrontal cortex function by a schizophrenia risk gene. Hum Brain Mapp.

[52] Nelson S.B., Valakh V. (2015). Excitatory/inhibitory balance and circuit homeostasis in autism spectrum disorders. Neuron.

[53] Marin O. (2012). Interneuron dysfunction in psychiatric disorders. Nat Rev Neurosci.

[54] Wilson T.W., Rojas D.C., Reite M.L., Teale P.D., Rogers S.J. (2007). Children and adolescents with autism exhibit reduced MEG steady-state gamma responses. Biol Psychiatry.

[55] Sun L., Grützner C., Bölte S., Wibral M., Tozman T., Schlitt S. (2012). Impaired gamma-band activity during perceptual organization in adults with autism spectrum disorders: Evidence for dysfunctional network activity in frontal-posterior cortices. J Neurosci.

[56] Peiker I., David N., Schneider T.R., Nolte G., Schöttle D., Engel A.K. (2015). Perceptual integration deficits in autism spectrum disorders are associated with reduced interhemispheric gamma-band coherence. J Neurosci.

[57] Dakin S., Frith U. (2005). Vagaries of visual perception in autism. Neuron.

[58] Khan S., Gramfort A., Shetty N.R., Kitzbichler M.G., Ganesan S., Moran J.M. (2013). Local and long-range functional connectivity is reduced in concert in autism spectrum disorders. Proc Natl Acad Sci U S A.

[59] Geschwind D.H., Levitt P. (2007). Autism spectrum disorders: Developmental disconnection syndromes. Curr Opin Neurobiol.

[60] Teipel S., Drzezga A., Grothe M.J., Barthel H., Chételat G., Schuff N. (2015). Multimodal imaging in Alzheimer’s disease: Validity and usefulness for early detection. Lancet Neurol.

[61] de Haan W., Stam C.J., Jones B.F., Zuiderwijk I.M., van Dijk B.W., Scheltens P. (2008). Resting-state oscillatory brain dynamics in Alzheimer disease. J Clin Neurophysiol.

[62] Tijms B.M., Wink A.M., de Haan W., van der Flier W.M., Stam C.J., Scheltens P., Barkhof F. (2013). Alzheimer’s disease: Connecting findings from graph theoretical studies of brain networks. Neurobiol Aging.

[63] Ranasinghe K.G., Hinkley L.B., Beagle A.J., Mizuiri D., Dowling A.F., Honma S.M. (2014). Regional functional connectivity predicts distinct cognitive impairments in Alzheimer’s disease spectrum. Neuroimage Clin.

[64] Hinkley L.B., Vinogradov S., Guggisberg A.G., Fisher M., Findlay A.M., Nagarajan S.S. (2011). Clinical symptoms and alpha band resting-state functional connectivity imaging in patients with schizophrenia: Implications for novel approaches to treatment. Biol Psychiatry.

[65] Petersen R.C., Doody R., Kurz A., Mohs R.C., Morris J.C., Rabins P.V. (2001). Current concepts in mild cognitive impairment. Arch Neurol.

[66] López M.E., Bruña R., Aurtenetxe S., Pineda-Pardo J.Á., Marcos A., Arrazola J. (2014). Alpha-band hypersynchronization in progressive mild cognitive impairment: A magnetoencephalography study. J Neurosci.

[67] Rossini P.M., Del Percio C., Pasqualetti P., Cassetta E., Binetti G., Dal Forno G. (2006). Conversion from mild cognitive impairment to Alzheimer’s disease is predicted by sources and coherence of brain electroencephalography rhythms. Neuroscience.

[68] Bajo R., Castellanos N.P., Cuesta P., Aurtenetxe S., Garcia-Prieto J., Gil-Gregorio P. (2012). Differential patterns of connectivity in progressive mild cognitive impairment. Brain Connect.

[69] Ahmadlou M., Adeli A., Bajo R., Adeli H. (2014). Complexity of functional connectivity networks in mild cognitive impairment subjects during a working memory task. Clin Neurophysiol.

[70] Dubarry A.S., Badier J.M., Trébuchon-Da Fonseca A., Gavaret M., Carron R., Bartolomei F. (2014). Simultaneous recording of MEG, EEG and intracerebral EEG during visual stimulation: From feasibility to single-trial analysis. Neuroimage.

[71] Esmail S., Linden D.E.J. (2014). Neural networks and neurofeedback in Parkinson’s disease. Neuroregulation.

[72] Hammond C., Bergman H., Brown P. (2007). Pathological synchronization in Parkinson’s disease: Networks, models and treatments. Trends Neurosci.

[73] Heinrichs-Graham E., Wilson T.W., Santamaria P.M., Heithoff S.K., Torres-Russotto D., Hutter-Saunders J.A. (2014). Neuromagnetic evidence of abnormal movement-related beta desynchronization in Parkinson’s disease. Cereb Cortex.

[74] Hall A.M., Roberson E.D. (2012). Mouse models of Alzheimer’s disease. Brain Res Bull.

[75] Elder G., Gama Sosa M., De Gasperi R. (2010). Transgenic mouse models of Alzheimer’s disease. Mt Sinai J Med.

[76] Busche M.A., Eichhoff G., Adelsberger H., Abramowski D., Wiederhold K.H., Haass C. (2008). Clusters of hyperactive neurons near amyloid plaques in a mouse model of Alzheimer’s disease. Science.

[77] Palop J.J., Mucke L. (2016). Network abnormalities and interneuron dysfunction in Alzheimer disease. Nat Rev Neurosci.

[78] Prvulovic D., Van de Ven V., Sack A.T., Maurer K., Linden D.E. (2005). Functional activation imaging in aging and dementia. Psychiatry Res.

[79] Lancaster T.M., Brindley L.M., Tansey K.E., Sims R.C., Mantripragada K., Owen M.J. (2015). Alzheimer’s risk variant in CLU is associated with neural inefficiency in healthy individuals. Alzheimers Dement.

[80] Stargardt A., Swaab D.F., Bossers K. (2015). Storm before the quiet: Neuronal hyperactivity and Aβ in the presymptomatic stages of Alzheimer’s disease. Neurobiol Aging.

[81] Kirov G., Pocklington A.J., Holmans P., Ivanov D., Ikeda M., Ruderfer D. (2012). De novo CNV analysis implicates specific abnormalities of postsynaptic signalling complexes in the pathogenesis of schizophrenia. Mol Psychiatry.

[82] Pocklington A.J., Rees E., Walters J.T., Han J., Kavanagh D.H., Chambert K.D. (2015). Novel findings from CNVs implicate inhibitory and excitatory signaling complexes in schizophrenia. Neuron.

[83] Jenkins J., Chow V., Blaskey L., Kuschner E., Qasmieh S., Gaetz L. (2016). Auditory evoked M100 response latency is delayed in children with 16p11.2 deletion but not 16p11.2 duplication. Cereb Cortex.

[84] Portmann T., Yang M., Mao R., Panagiotakos G., Ellegood J., Dolen G. (2014). Behavioral abnormalities and circuit defects in the basal ganglia of a mouse model of 16p11.2 deletion syndrome. Cell Rep.

[85] Koutsouleris N., Meisenzahl E.M., Davatzikos C., Bottlender R., Frodl T., Scheuerecker J. (2009). Use of neuroanatomical pattern classification to identify subjects in at-risk mental states of psychosis and predict disease transition. Arch Gen Psychiatry.

[86] Clementz B.A., Sweeney J.A., Hamm J.P., Ivleva E.I., Ethridge L.E., Pearlson G.D. (2016). Identification of distinct psychosis biotypes using brain-based biomarkers. Am J Psychiatry.

[87] Deco G., Ponce-Alvarez A., Hagmann P., Romani G.L., Mantini D., Corbetta M. (2014). How local excitation-inhibition ratio impacts the whole brain dynamics. J Neurosci.

[88] Stephan K.E., Iglesias S., Heinzle J., Diaconescu A.O. (2015). Translational perspectives for computational neuroimaging. Neuron.

[89] Schneiderman J.F. (2014). Information content with low- vs. high-T(c) SQUID arrays in MEG recordings: The case for high-T(c) SQUID-based MEG. J Neurosci Methods.

[90] Oostenveld R., Fries P., Maris E., Schoffelen J.M. (2011). FieldTrip: Open source software for advanced analysis of MEG, EEG, and invasive electrophysiological data. Comput Intell Neurosci.

[91] Tadel F., Baillet S., Mosher J.C., Pantazis D., Leahy R.M. (2011). Brainstorm: A user-friendly application for MEG/EEG analysis. Comput Intell Neurosci.

[92] Gross J., Baillet S., Barnes G.R., Henson R.N., Hillebrand A., Jensen O. (2013). Good practice for conducting and reporting MEG research. Neuroimage.

[93] Troebinger L., López J.D., Lutti A., Bradbury D., Bestmann S., Barnes G. (2014). High precision anatomy for MEG. Neuroimage.

[94] Troebinger L., López J.D., Lutti A., Bestmann S., Barnes G. (2014). Discrimination of cortical laminae using MEG. Neuroimage.

[95] Neuling T., Ruhnau P., Fuscà M., Demarchi G., Herrmann C.S., Weisz N. (2015). Friends, not foes: Magnetoencephalography as a tool to uncover brain dynamics during transcranial alternating current stimulation. Neuroimage.

[96] Keitel A., Gross J. (2016). Individual human brain areas can be identified from their characteristic spectral activation fingerprints. PLoS Biol.

[97] Myers N.E., Rohenkohl G., Wyart V., Woolrich M.W., Nobre A.C., Stokes M.G. (2015). Testing sensory evidence against mnemonic templates. Elife.

